# Knowledge Gaps Regarding Overweight and Obesity in Pregnancy: A Cross-Sectional Study Among Polish Women

**DOI:** 10.3390/nu18020203

**Published:** 2026-01-08

**Authors:** Anita Froń, Magdalena Orczyk-Pawiłowicz

**Affiliations:** Division of Chemistry and Immunochemistry, Department of Biochemistry and Immunochemistry, Wroclaw Medical University, M. Skłodowskiej-Curie 48/50, 50-369 Wroclaw, Poland

**Keywords:** maternal overweight, maternal obesity, breastfeeding, perinatal health, health knowledge, health literacy

## Abstract

**Background:** Maternal overweight and obesity, which show a rising trend globally, are associated with adverse pregnancy outcomes and long-term health risks for both mother and child. Awareness and understanding of these risks among women of reproductive age are essential for effective prevention and early intervention. **Methods:** We conducted a cross-sectional survey among 958 women planning pregnancy, currently pregnant or breastfeeding to assess their knowledge and attitudes regarding overweight and obesity in the perinatal period. The questionnaire covered lifestyle behaviors, breastfeeding practices, and knowledge related to overweight and obesity in pregnancy. **Results:** Overall knowledge regarding the consequences of maternal overweight and obesity was low, with notable deficits in understanding the associated health risks and frequent misconceptions about dietary recommendations during pregnancy. Awareness gaps were particularly noticeable in domains related to fetal outcomes and recommended energy requirements across pregnancy. Excessive gestational weight gain was reported in over 75% of pregnancies, including among women with normal body mass index. Participation in antenatal classes, current breastfeeding and older age were significantly associated with higher knowledge; however, these factors together explained only 6.2% of variability. Still, several key aspects were not well recognized despite high educational attainment and frequent contact with maternity care services. **Conclusions:** Our study highlights a clear and urgent need for better, more targeted educational strategies to improve women’s understanding of metabolic health and nutrition before and during pregnancy. The low explained variance indicates that maternal knowledge is influenced by multifactorial and not easily captured determinants, emphasizing the need for comprehensive and individualized educational approaches. Enhancing maternal awareness could support better health outcomes for both mothers and their offspring.

## 1. Introduction

Maternal overweight and obesity represent a growing public health concern worldwide and are strongly associated with an increased risk of adverse pregnancy and neonatal outcomes, including gestational diabetes mellitus (GDM), hypertension, cesarean delivery, and long-term cardiometabolic complications in both mother and child [1,2,3]. In Poland, recent epidemiological data from 2022 indicate that 42.6% of adult women aged ≥20 years are overweight and 11.7% are obese, confirming that excess body weight is highly prevalent among women of reproductive age [4]. Furthermore, national health reports show a steadily rising trend in obesity among women in the reproductive period over the last decade [5]. Projections for 2020–2035 estimate an annual increase of approximately 1.7% in adult obesity rates in Poland, with projections suggesting that nearly one-third of Polish women may be obese by 2035 [6].

Standardized body mass index (BMI) categories, established by the World Health Organization classify BMI values as <18.5 kg/m^2^ (underweight), 18.5–24.9 kg/m^2^ (normal weight), 25.0–29.9 kg/m^2^ (overweight), and ≥30.0 kg/m^2^ (obesity), with BMI calculated as weight (kg) divided by height squared (m^2^) [7]. In obstetric practice, maternal obesity is diagnosed when a woman’s BMI in early pregnancy—typically assessed at the first antenatal visit—measures 30 kg/m^2^ or higher [5].

However, despite the growing prevalence of overweight and obesity, many women remain unaware of their own weight status, the appropriate gestational weight gain (GWG), or the potential implications for maternal and neonatal health. In several cohorts, the majority of pregnant women were unable to correctly identify the recommended GWG, with nearly 60% providing values inconsistent with clinical guidelines [8]. At the same time, 74% of women with obesity underestimated their BMI category, and a considerable proportion overestimated their recommended GWG—64% among women with obesity and 40% among those with overweight [9]. These misperceptions extend beyond weight assessment alone, as women often demonstrate limited knowledge of the pregnancy complications associated with excess maternal weight as well as the long-term consequences for the child [10,11].

Recent research also highlights that excess maternal weight rarely occurs in isolation [12]. In a large cohort of expectant parents, 62% of couples had at least one partner with overweight or obesity, and nearly one third of women entered pregnancy with elevated weight. Importantly, the likelihood of maternal obesity increased over six-fold when the partner was also obese, demonstrating a strong interpersonal component in weight-related behaviors [13].

The rising prevalence of overweight and obesity among women of reproductive age underscores the need for healthcare systems to be adequately prepared—both in terms of right clinical skills and resources—to provide safe and effective care for pregnant women with obesity throughout pregnancy and childbirth [5]. Preconception care plays a critical role in this context and should include education on nutrition, weight management, and the prevention of non-communicable diseases [14]. Even modest improvements in pre-pregnancy weight can significantly influence reproductive outcomes; for example, reducing BMI by just one unit or achieving a 10% weight loss has been shown to significantly increase the likelihood of conception and reduce the risk of complications for both mother and fetus [5]. Despite these recommendations, most women report receiving little or no counselling regarding appropriate gestational weight gain or the health consequences associated with excess maternal weight [15,16]. This gap in routine antenatal guidance persists even though lifestyle interventions, particularly healthy eating with defined caloric and macronutrient goals, have been shown to effectively limit excessive GWG among overweight and obese pregnant women [17].

Importantly, maternal knowledge should be understood as an important component of health-related decision-making, but not a sole determinant. Health behaviors during pregnancy are shaped by awareness of risks as well as psychological factors, social context, healthcare communication, and structural constraints within maternity care [18,19,20,21].

Paradoxically, women with a low or normal BMI are no more likely to receive professional advice on healthy body weight or lifestyle before or during pregnancy, whereas those with overweight or obesity are more frequently counselled on these issues [11]. This pattern suggests that preventive strategies are often reactive rather than proactive, and that opportunities for early intervention—ideally before conception—remain not fully leveraged.

Maternal overweight and obesity have well-established effects on lactation outcomes. Women with higher pre-pregnancy BMI are more likely to experience delayed onset of lactogenesis, lower breastfeeding initiation rates, and earlier cessation of breastfeeding [22,23]. Moreover, maternal adiposity influences the immunological composition of breast milk, including concentrations of adipokines and inflammatory markers, which is particularly relevant in the context of metabolic health [24,25]. Importantly, breastfeeding is one of the key modifiable factors that can attenuate the adverse metabolic programming associated with maternal obesity [26,27,28].

Although several studies have examined women’s knowledge of gestational diabetes [29,30], breastfeeding [31,32,33], or lifestyle recommendations during pregnancy [34,35,36,37], there is a striking lack of research assessing women’s awareness of the risks associated with maternal overweight and obesity in Poland. The only available Polish study [38] remains a preprint and has not been peer-reviewed, reflecting the absence of validated, high-quality national data. This emphasizes the need for robust, population-based research such as the present study.

Given the rising burden of maternal overweight and obesity and the lack of comprehensive national data, this study aimed to evaluate Polish women’s awareness of obesity-related risks in pregnancy and to examine which sociodemographic, obstetric and other factors influence their level of knowledge.

## 2. Materials and Methods

### 2.1. Study Design

This was a nationwide, cross-sectional survey study conducted between December 2024 and May 2025. The study was designed to assess women’s knowledge regarding overweight and obesity during pregnancy and breastfeeding. The questionnaire (https://forms.gle/VuQjNJi5BsTRGgYU6 (accessed on 29 December 2025)) consisted of four sections: sociodemographic data, obstetric data, knowledge regarding overweight and obesity in pregnancy, and knowledge regarding breastfeeding, comprising a total of 39 questions. Most questions were single- or multiple-choice, with a few allowing open-ended responses. The survey was anonymous and administered online using Google Forms. Prior to data collection, the questionnaire was pilot-tested, and feedback was incorporated to refine the final version.

### 2.2. Study Sample

The target population included women aged 18–45 years who were planning a pregnancy, currently pregnant, or had previously given birth. According to Statistics Poland (GUS) [39], the female population aged 18–45 years in 2024 was approximately 7.5 million, which constituted the source population for this study.

The minimum required sample size was calculated using a 95% confidence level, 5% margin of error, and a response distribution of 50%, which indicated that at least 384 participants were needed. In total, 958 women completed the survey, exceeding the required sample size and ensuring sufficient statistical power.

### 2.3. Recruitment

Participants were recruited nationwide through local parenting and pregnancy-related groups on Facebook. The survey link was publicly available and participation was voluntary. Eligibility criteria were provided at the beginning of the survey, and informed consent was obtained electronically prior to participation. The online survey design and recruitment via social media platforms enabled broad nationwide reach but may have introduced selection bias toward women who are more health-conscious and digitally active.

### 2.4. Ethics Statement

The study was conducted in accordance with the Declaration of Helsinki. Participation was voluntary, and informed consent was obtained from all participants at the beginning of the questionnaire. The survey was fully anonymous and did not collect any identifiable personal data. According to local regulations and institutional policies, anonymous, non-interventional survey studies based exclusively on non-identifiable data are exempt from ethics committee review. Therefore, ethical approval was not required for this study.

### 2.5. Questionnaire Development

The details concerning the assessment of knowledge levels were adapted from previous studies [29,40] and modified. For single-choice questions, responses were scored as follows: 2 points—correct answer, 1 point—“don’t know”/“not sure”, 0 points—incorrect answer. For multiple-choice questions, each correct option selected was awarded 1 point. If the respondent provided an additional correct answer in the open-ended “other” field, this was also awarded 1 point. The total knowledge score was calculated as the sum of all points obtained across the questionnaire. The following evaluation scale of grading was used: poor (<60%), moderate (60–79%), and detailed (80–100%). Although the primary focus of the questionnaire was maternal overweight and obesity, questions regarding breastfeeding benefits were included due to the well-established association between maternal metabolic status, breastfeeding initiation and duration, and long-term metabolic outcomes in the offspring. At the time of study design, no validated, standardized instrument was available that comprehensively assessed women’s knowledge regarding maternal overweight and obesity during pregnancy in conjunction with breastfeeding-related metabolic implications. Therefore, a study-specific questionnaire was developed based on existing literature, which may limit direct comparability with studies.

### 2.6. Statistical Analysis

Descriptive statistics were used to summarize participants’ sociodemographic characteristics, lifestyle factors, and knowledge scores. Continuous variables were presented as means and standard deviations (SD), while categorical variables were reported as frequencies and percentages. Because knowledge scores showed deviations from normal distribution and several categorical predictors had highly unequal group sizes, nonparametric tests were applied. Group differences were assessed using the Kruskal–Wallis test with Dwass–Steel–Critchlow–Fligner post hoc comparisons, and two-group comparisons with the Brunner–Munzel test. Associations between continuous variables were examined using Spearman’s rank correlation. The relationship between pre-pregnancy BMI category and gestational weight gain adequacy was examined using a chi-square test, with effect size expressed as Cramer’s V. To identify independent predictors of maternal knowledge, a multivariable linear regression model was constructed. Model fit was assessed using R^2^ and adjusted R^2^ values. To clarify the assumed causal relationships between sociodemographic factors, reproductive characteristics, healthcare exposure, and maternal knowledge, a directed acyclic graph (DAG) was constructed to guide confounder selection and interpretation of the multivariable model (see Supplementary Figure S1).

All analyses were performed using Jamovi (The Jamovi Project, 2025) (version 2.6.45) [41].

### 2.7. The Sociodemographic Data

The first section collected sociodemographic information. Participants were asked about their current reproductive status (planning pregnancy, being pregnant for the first time, already a mother—breastfeeding or not, or being in a subsequent pregnancy—breastfeeding or not), as well as their age, marital status, employment situation (e.g., studying, employed, self-employed, on maternity leave, unemployed), and monthly net household income per capita. Income was categorized into five ranges (<3000 PLN, 3000–4499 PLN, 4500–5999 PLN, 6000–10,000 PLN, and >10,000 PLN), established with reference to both national quintile distribution of disposable household income reported by GUS [42] and recent increases in the minimum wage in Poland [43]. The threshold of 3000 PLN was set close to the level of the statutory minimum wage (net), thereby separating women with incomes below the minimum wage from those with higher earnings. Higher brackets reflected middle- and upper-income categories in the Polish socioeconomic context. Place of residence was also assessed, distinguishing between rural areas, towns with fewer than 50,000 inhabitants, towns with 50,000–150,000 inhabitants, towns with 150,000–500,000 inhabitants, and large cities with more than 500,000 inhabitants. Education level was categorized into four groups: primary, vocational, secondary, and higher education. To facilitate statistical analysis, the study population was categorized into five age groups: 18–25, 26–30, 31–35, 36–40, and 41–45 years.

### 2.8. The Obstetric Data

The second section focused on obstetric data. Participants reported the number of pregnancies, their outcomes (vaginal delivery, elective or emergency cesarean section, miscarriage, preterm or term birth), and difficulties with conception. Further questions explored breastfeeding practices, such as intention to breastfeed, reasons for not breastfeeding (including insufficient milk supply, health-related contraindications, occupational demands, or previous negative experiences), duration of breastfeeding for each child, and whether breastfeeding was exclusive or combined with expressed milk. Additional items addressed pre-pregnancy BMI, gestational weight gain according to Institute of Medicine recommendations [44], physical activity during pregnancy, participation in antenatal classes, and lifestyle behaviors such as alcohol consumption, smoking, and the use of medications or supplements (e.g., folic acid, vitamin D, pregnancy supplements). Pre-pregnancy BMI and gestational weight gain were self-reported and may be subject to recall and reporting bias, which was considered at the study design stage.

### 2.9. The Knowledge Regarding Overweight and Obesity in Pregnancy

The third section assessed women’s knowledge regarding overweight and obesity in pregnancy. Respondents were asked to identify health risks linked to maternal overweight and obesity, both for the mother (e.g., hypertension, diabetes, preeclampsia, edema, varicose veins, constipation) and for the child (e.g., preterm birth, macrosomia, congenital anomalies, growth restriction, shoulder dystocia, or higher long-term risk of obesity and metabolic disorders). To evaluate nutritional knowledge, the questionnaire included items on how caloric requirements change across trimesters, whether dieting is recommended during pregnancy, and from which sources they obtain health information (e.g., gynecologist, midwife, antenatal classes, family, internet, or books). It also captured whether women had received dietary and physical activity advice during pregnancy from healthcare providers.

### 2.10. The Knowledge Regarding Breastfeeding

The final section explored knowledge related to breastfeeding. It included questions on whether women had been informed by healthcare professionals about the health benefits of breastfeeding for both the child and the mother. Participants identified perceived benefits of breastfeeding, which covered both child-related outcomes (e.g., cognitive development, stronger immunity, lower risk of obesity and metabolic diseases) and maternal outcomes (e.g., reduced risk of breast and ovarian cancer). Additional items examined attitudes toward breastfeeding in overweight or obese women and awareness of the concept of metabolic programming, i.e., whether infant feeding during the first six months of life may influence long-term health outcomes.

## 3. Results

### 3.1. Sociodemographic Variables

The study included 958 women aged 18 to 45 years. The largest subgroup consisted of women aged 30 years (*n* = 96). The mean age of the participants was 32.2 ± 4.7 years, while the median age was 28.5 years.

Almost one-third of respondents lived in large cities with more than 500,000 inhabitants (31.6%), followed by rural areas (24.0%) and medium-sized cities of 150,000–499,999 residents (17.3%). Smaller towns with fewer than 50,000 inhabitants accounted for 12.8% of the sample, while 14.2% lived in towns with 50,000–149,999 residents.

The majority of participants reported being married (761; 79.4%), while 16.4% were in an informal relationship (*n* = 157) and 3.1% were single (*n* = 30). Only a small proportion were divorced or separated (*n* = 9; 0.9%) or widowed (*n* = 1; 0.1%).

Regarding educational attainment, most respondents had higher education (815; 85.1%), followed by secondary education (130; 13.6%). Vocational education was reported by 1.0% of participants (*n* = 10), and only 0.3% had primary education (*n* = 3) (Table 1).

### 3.2. Pregnancy and Delivery Characteristics

Over half of the respondents were already mothers and currently breastfeeding (498; 52.0%). An additional 25.2% (*n* = 241) were mothers who were not breastfeeding at the time of the survey. Women in a subsequent pregnancy accounted for 9.1% of the sample (*n* = 87), and 2.9% (*n* = 28) were in a subsequent pregnancy while also currently breastfeeding. A smaller proportion of participants were planning a pregnancy (53; 5.5%) or were in their first pregnancy (51; 5.3%).

The distribution of previous pregnancies showed that nearly half of the respondents had experienced one pregnancy (427; 44.6%), while 31.0% had two pregnancies (297). A smaller proportion reported three (117; 12.2%) or four pregnancies (47; 4.9%). Nulligravid women accounted for 5.4% of the sample (52 participants). Only 1.9% of respondents (*n* = 18) reported having five or more previous pregnancies. The median number of pregnancies in the study population was 1.5. Difficulties conceiving a child were reported by 29.2% of participants (*n* = 280).

The cohort population consisted of 958 women, including 53 planning a pregnancy (one with a prior miscarriage). Participants also detailed the outcomes of their past pregnancies. As a result, the total number of pregnancy outcomes exceeded the number of respondents.

A small subset of women entered incomplete or internally inconsistent data for gestational age or mode of delivery (e.g., indicating a delivery type without gestational age, or vice versa). Such cases were not excluded from the study; instead, missing components were coded as missing data (NA) and handled using a complete-case approach for each specific analysis.

Among all reported pregnancy outcomes (*n* = 1218), the majority ended with a vaginal delivery (734 cases). Emergency cesarean section was performed in 284 pregnancies, while 200 cases were planned cesarean section.

Regarding the timing of pregnancy outcomes (*n* = 878), 215 pregnancies ended before 22 weeks of gestation (miscarriages). Most deliveries occurred between 37 and 40 weeks (*n* = 370), while 279 women gave birth between 40 and 42 weeks of gestation. Only a small proportion of pregnancies continued beyond 42 weeks (*n* = 14) (Table 2).

### 3.3. Maternal Weight Status

Regarding pre-pregnancy body weight among women who had ever been pregnant (*n* = 906), the majority reported having a normal BMI before pregnancy (557; 61.5%). Excess body weight was common, with 188 women (20.8%) indicating overweight and 109 (12.0%) obesity. A smaller proportion of respondents entered pregnancy underweight (52; 5.7%).

Among the 297 women who reported having overweight or obesity before at least one pregnancy, most indicated this prior to one pregnancy only (173; 58.2%). Overweight or obesity before two pregnancies was declared by 88 women (29.6%), and before three pregnancies by 26 women (8.8%). A smaller proportion experienced overweight or obesity before four (*n* = 9; 3.0%) or five pregnancies (*n* = 1; 0.3%).

Information regarding gestational weight gain adequacy was available for 1284 pregnancies, reflecting minor discrepancies arising from incomplete or irregular reporting across individual pregnancy fields. Among these, adequate gestational weight gain was indicated for 317 pregnancies (24.7%), whereas 967 pregnancies (75.3%) were characterized by excessive weight gain (Table 3).

To explore whether pre-pregnancy BMI was associated with gestational weight gain adequacy, a cross-tabulation was generated comparing BMI categories with GWG outcomes across pregnancies. Because many participants reported multiple pregnancies, only the first pregnancy per respondent was included in this analysis to avoid non-independence of observations. Among the 849 pregnancies with available data, excessive GWG was common across all BMI groups, including women with normal BMI (111/523; 21.2%), overweight (67/177; 37.9%), and obesity (28/102; 27.5%) (Table 4).

There was a statistically significant association between pre-pregnancy BMI category and GWG adequacy (χ^2^(3) = 26.9, *p* < 0.001, Cramer’s V = 0.18). Excessive GWG was most frequent among women with pre-pregnancy overweight (37.9%), followed by those with obesity (27.5%) and normal weight (21.2%), and least common among underweight women (8.5%).

### 3.4. Management of Pregnancy

During their pregnancies, most respondents reported engaging in physical activity (573 out of 903 valid answers; 63.4%). In contrast, 330 women (36.6%) stated that they were not physically active during pregnancy. The majority attended antenatal classes (609; 67.2%).

None of the participants reported regular alcohol use during pregnancy. Almost all women declared complete abstinence, while only two participants (0.2%) mentioned occasional alcohol consumption. Occasional smoking was declared by 11 women (1.2%), while 14 participants (1.5%) reported smoking regularly during pregnancy.

Supplement and medication use was also assessed. A total of 840 respondents indicated using pregnancy-specific supplements. Folic acid supplementation was declared by 691 women, and vitamin D by 549. Participants commonly took thyroid medications (*n* = 294), antibiotics (*n* = 161), treatment for urinary or reproductive tract infections (*n* = 159 and 163, respectively), prophylactic anticoagulation therapy (*n* = 153), antihypertensive agents (*n* = 89), and medications for insulin resistance or diabetes (*n* = 98). A smaller number of respondents used antiallergic drugs (*n* = 45), antidepressants (*n* = 23), or other medications not listed (*n* = 99) (Supplementary Table S1).

### 3.5. Intention to Breastfeed and Breastfeeding Duration

When asked whether they intended to breastfeed or had previously breastfed their child, the vast majority of respondents answered affirmatively. A total of 904 women (94.4%) reported that they either planned to breastfeed or had already done so. Only 39 respondents (4.1%) stated that they did not intend to breastfeed, while 15 women (1.6%) had not yet made a decision.

Among mothers who were no longer breastfeeding at the time of the survey (*n* = 693), the reported duration of breastfeeding varied widely. A small proportion breastfed for 1 month or less (*n* = 50; 7.2%), while slightly larger groups continued for 2–3 months (*n* = 60; 8.7%) or 4–6 months (*n* = 63; 9.1%). Approximately one in seven women breastfed for 7–12 months (*n* = 98; 14.1%). Longer breastfeeding durations were also common: 226 respondents (32.6%) breastfed for over one year, and 196 women (28.3%) reported breastfeeding for more than two years (Table 5).

Among respondents who were breastfeeding at the time of the survey, the duration of breastfeeding showed variability. Reported durations ranged from 0.25 to 60 months. The median breastfeeding period was 8 months, with an interquartile range (IQR) of 4–17 months, indicating that half of the currently nursing women had been breastfeeding for between four and seventeen months. The mean duration was 11.7 months, reflecting the influence of several longer breastfeeding periods extending beyond two years. Additionally, 13 women reported tandem nursing.

Among respondents who did not or do not want to breastfeed, the most frequently declared reason was insufficient milk supply (*n* = 16). Several women indicated lack of desire to breastfeed (*n* = 8), while others cited medical contraindications (*n* = 4) or previous negative breastfeeding experiences (*n* = 4).

Individual narratives also appeared: one participant described having to stop breastfeeding due to antibiotic treatment related to poor cesarean wound healing, and another linked cessation to stress after an infant choking episode followed by hospitalization and subsequent COVID-19 infection, after which lactation declined. One respondent expressed the belief that breastfeeding is not necessarily the best option for all infants.

### 3.6. Women’s Knowledge Regarding Overweight and Obesity in Pregnancy

Most respondents declared that they were aware of how maternal overweight or obesity may influence the course of pregnancy. A total of 711 women (74.3%) answered that they were familiar with these effects, while 90 (9.4%) reported not knowing them, and 157 (16.4%) selected “uncertain” (Supplementary Figure S2).

**Figure 1:**
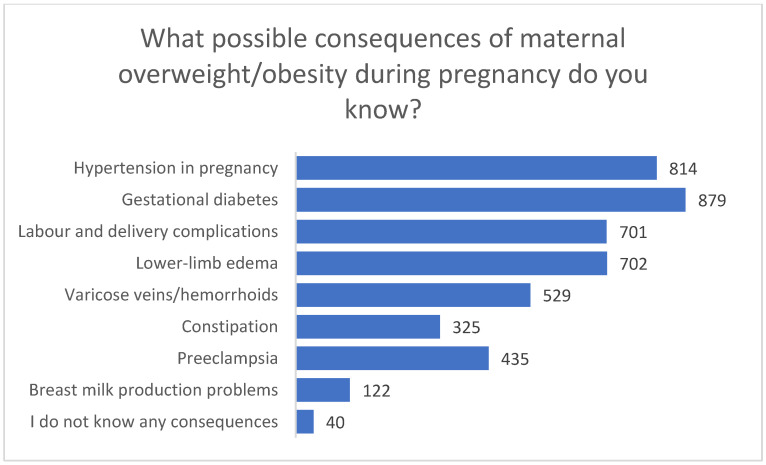
Perceived maternal consequences of overweight/obesity during pregnancy (multiple responses allowed).

Regarding perceived maternal complications associated with overweight or obesity in pregnancy, the most frequently indicated consequences were gestational diabetes (*n* = 879) and gestational hypertension (*n* = 814). Other commonly reported risks included edema of the lower limbs (*n* = 702), delivery complications (*n* = 701), varicose veins or hemorrhoids (*n* = 529), and pre-eclampsia (*n* = 435). Constipation (*n* = 325) and difficulties with milk production (*n* = 122) were mentioned less often. Only 40 respondents stated that they did not know any maternal complications. In the open-ended field, one participant additionally indicated “back problems” (Figure 1).

Participants also identified several potential risks for the child. The most frequently reported were fetal macrosomia (*n* = 649) and an increased likelihood of metabolic complications in the offspring, such as insulin resistance or type 2 diabetes (*n* = 609). Many respondents also mentioned a higher risk of childhood or adult overweight/obesity (*n* = 556), shoulder dystocia (*n* = 246), prematurity (*n* = 420), congenital anomalies (*n* = 158), and fetal growth restriction (*n* = 145). A smaller proportion of women indicated post-term birth as a possible consequence (*n* = 132). A total of 160 participants reported no knowledge of any consequences.

**Figure 2:**
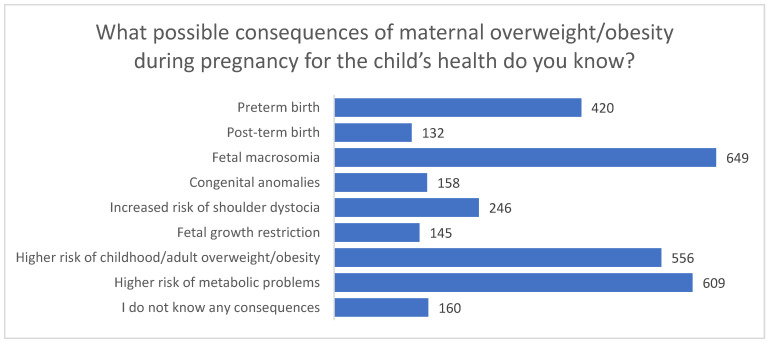
Perceived child health risks associated with maternal overweight/obesity during pregnancy (multiple responses allowed).

In the free-text responses, one participant mentioned “a higher percentage of autism”, and another stated that “there is no evidence that the above outcomes are caused by overweight”, although these comments were not repeated by other respondents (Figure 2). One participant provided the same additional open-ended comment in both questions regarding maternal and child health risks, stating that “assessments of my health based on BMI are not reliable; I believe that factors other than BMI are more accurate predictors of risks and outcomes.”

Awareness concerning changes in energy requirements during pregnancy varied considerably among respondents. In the first trimester, 307 women (34.0%) correctly indicated that energy requirements do not increase, while 140 (15.5%) believed the increase to be around 80 kcal and 159 (17.6%) selected 150 kcal. Over one-third (*n* = 352; 39.0%) reported not knowing the recommended values (Supplementary Figure S3A).

Knowledge regarding caloric requirements was similarly inconsistent for subsequent trimesters. For the second trimester, 362 women (40.0%) correctly selected the recommended increase of approximately 260 kcal, whereas 157 (17.3%) and 20 (2.2%) chose 360 kcal and 460 kcal, respectively. A substantial proportion (*n* = 419; 46.3%) reported not knowing the answer (Supplementary Figure S3B).

In the third trimester, 391 respondents (43.1%) correctly indicated an increase of around 475 kcal, while 125 (13.8%) and 18 (2.0%) selected higher values. Again, a large share (*n* = 424; 46.1%) were unsure (Supplementary Figure S3C).

When asked whether weight reduction is advisable during pregnancy, 428 women (46.6%) correctly stated that intentional weight loss is not recommended, whereas 40 (4.4%) believed it is safe, and 277 (30.2%) indicated that weight reduction may be acceptable only until achieving a normal BMI. The remaining 213 (23.2%) were unsure (Supplementary Figure S4).

Respondents reported multiple information channels regarding health in pregnancy. The most frequently indicated sources were the internet (*n* = 811), obstetrician-gynecologists (*n* = 733), books and magazines (*n* = 495), midwives (*n* = 462), and antenatal classes (*n* = 452). Family and friends were mentioned less often (*n* = 234), and only a very small number of respondents (*n* = 6) stated that they were not interested in this topic (Supplementary Figure S5).

After excluding respondents who indicated that the question did not apply to them (*n* = 59), data were analyzed for 899 women who had experienced at least one pregnancy. Dietary counselling during pregnancy was reported by 347 respondents (38.6%), whereas 507 (56.4%) stated they did not receive such advice and 45 (5.0%) could not recall it. Similarly, 399 women (44.4%) stated that they had received recommendations regarding physical activity during pregnancy, while 445 (49.5%) indicated they had not and 55 (6.1%) were unsure (Supplementary Table S2).

### 3.7. Women’s Knowledge Regarding Breastfeeding

Among respondents with previous pregnancy experience, 622 women (69.5%) reported having been informed by a physician or midwife about the health benefits of breastfeeding for the child. A further 225 (25.1%) indicated that they had not received such information, while 48 (5.4%) did not remember. Regarding maternal health benefits, 479 participants (53.5%) stated that they had been informed about these advantages, 352 (39.3%) denied receiving such guidance, and 65 (7.3%) were unsure (Supplementary Table S3). These items were answered only by women who had previously been pregnant or had already discussed breastfeeding with a provider; therefore, denominators are lower and vary due to missing responses.

When asked whether women with overweight or obesity can breastfeed, nearly all participants expressed a positive view: 821 women (85.7%) answered “definitely yes,” 131 (13.7%) “rather yes,” while only 4 (0.4%) and 2 (0.2%) selected “rather no” and “definitely no,” respectively (Supplementary Figure S6).

Respondents identified numerous health benefits associated with breastfeeding. The most frequently indicated advantages included strengthening the emotional bond between mother and child (914 responses) and enhancing the infant’s immune system (923 responses). Many participants also recognized its positive influence on the child’s intellectual development (666 responses), reduced risk of respiratory diseases (*n* = 574), decreased likelihood of developing diabetes (*n* = 592), and lower risk of childhood overweight and obesity (*n* = 619).

**Figure 3:**
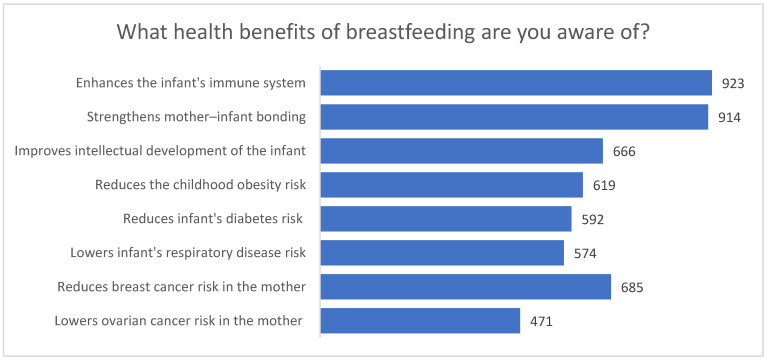
Health benefits of breastfeeding identified by respondents (multiple responses allowed).

Additionally, women were aware of maternal health benefits, such as a reduced risk of breast cancer (685 responses) and ovarian cancer (471 responses) (Figure 3). Only a very small number of respondents (*n* = 3) reported that they did not know any benefits related to breastfeeding.

Additionally, women provided several free-text answers regarding perceived benefits of breastfeeding. The most frequently mentioned advantages included practical aspects such as convenience, constant availability of milk, and lower costs (*n* = 9). Some of them highlighted the potential for faster postpartum weight reduction (*n* = 5), as well as a positive impact on craniofacial and jaw development (*n* = 3). Other comments referred to reduced risk of postpartum depression (*n* = 2), accelerated uterine involution (*n* = 2), the backwash effect (*n* = 1) and lower risk of food allergies (*n* = 1). A few respondents expressed critical or alternative perspectives. One participant explicitly disagreed with several listed benefits, emphasizing that strong maternal-infant bonding can also be achieved with formula feeding. Another woman stressed that, in her view, healthy family nutrition and lifestyle habits are more important predictors of health than BMI or calorie-based measures, indicating broader skepticism toward weight-focused counselling.

Moreover, participants were asked whether, in their opinion, infant feeding in the first six months of life has an impact on later development, including metabolic programming. Most respondents (*n* = 820; 85.6%) agreed, whereas 36 (3.8%) did not perceive such an association and 102 (10.6%) reported uncertainty (Supplementary Figure S7).

### 3.8. Knowledge Level of the Study Population

**Figure 4:**
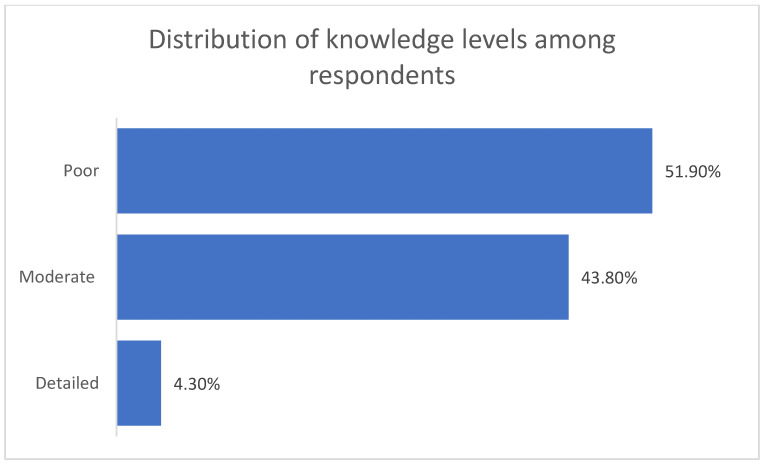
Knowledge level categories in the study population.

Based on the applied scoring system, the majority of respondents demonstrated a low level of knowledge regarding the impact of maternal overweight and obesity on pregnancy and infant outcomes. More than half of the participants were classified as having poor knowledge (497/958; 51.9%). A further 43.8% (420/958) presented a moderate level of knowledge. Only a small proportion of the sample achieved a detailed level of knowledge (41/958; 4.3%). The mean overall knowledge score in the study population was 57.6%, with values ranging from 18.4% to 89.5%. This indicates a wide variability in respondents’ awareness regarding the impact of maternal overweight and obesity on pregnancy outcomes and infant health (Figure 4).

### 3.9. Impact of Educational Level on Knowledge Scores

There were statistically significant differences in knowledge scores across educational groups (Kruskal–Wallis χ^2^ = 33.7, *df* = 3, *p* < 0.001, ε^2^ = 0.035), suggesting a small effect of education on overall knowledge.

Participants with higher education achieved the highest knowledge scores (mean 58.6%, median 60.5%), followed by those with secondary education (53.0%, median 52.6%). Women with primary education demonstrated moderate knowledge levels (mean 50.0%, median 47.4%), whereas the lowest scores were observed among women with vocational education (38.2%, median 43.4%) (Table 6).

Pairwise post hoc DSCF comparisons showed that women with higher education scored significantly higher than those with secondary (W = −5.973, *p* < 0.001) and vocational education (W = −5.627, *p* < 0.001). Women with secondary education also scored higher than those with vocational education (W = 4.201, *p* = 0.016). No significant differences were observed between the primary education group and any other category (*p* > 0.05), although this subgroup was very small (*n* = 3), limiting interpretability.

### 3.10. Impact of Antenatal Class Attendance on Knowledge Scores

There was a statistically significant difference in knowledge scores between women who attended antenatal classes and those who did not. The non-parametric Kruskal–Wallis test showed that participants who attended antenatal classes achieved significantly higher knowledge scores (χ^2^ = 17.4, *df* = 1, *p* < 0.001, ε^2^ = 0.019). On average, these women achieved a mean score of 59.0%, compared with 55.2% among those who did not participate (Table 6).

The Brunner–Munzel test confirmed this difference (BM statistic = 4.20, *df* = 545, *p* < 0.001). The relative effect was 0.586 (95% CI: 0.546–0.626), indicating that a randomly selected woman who attended antenatal classes had a 58.6% probability of achieving a higher knowledge score than a woman who did not attend.

These findings indicate that participation in antenatal education is associated with modestly improved knowledge regarding the impact of maternal overweight and obesity on pregnancy and infant outcomes.

### 3.11. Knowledge Level in Relation to Reproductive Status

There were statistically significant differences in knowledge scores across reproductive-status groups (Kruskal–Wallis χ^2^ = 20.0, *df* = 3, *p* < 0.001), with a small effect size (ε^2^ = 0.0209). This indicates that reproductive experience was only weakly associated with the level of maternal knowledge regarding overweight/obesity in pregnancy.

Women who were already mothers and currently breastfeeding achieved the highest knowledge scores (mean 59.2%). Their results were followed by mothers who were no longer breastfeeding (mean 56.2%). Participants who were pregnant for the first time demonstrated slightly lower knowledge (mean 54.3%), while the lowest scores were observed among women planning pregnancy (mean 53.0%) (Table 6).

DSCF post hoc revealed that two contrasts reached statistical significance:

- Mothers who were breastfeeding scored higher than mothers who were not breastfeeding (W = 4.664, *p* = 0.005),
- Mothers who were breastfeeding also scored higher than women planning pregnancy (W = 4.286, *p* = 0.013).

No other pairwise differences were statistically significant.

Overall, the results suggest a gradual increase in knowledge with increasing reproductive experience, particularly among women who have previously breastfed.

### 3.12. Maternal Knowledge in Relation to Pre-Pregnancy BMI

There were no statistically significant differences in knowledge scores across pre-pregnancy BMI categories (Kruskal–Wallis χ^2^ = 8.51, *df* = 4, *p* = 0.075), suggesting that BMI status before pregnancy was not associated with the level of knowledge regarding the impact of overweight and obesity on pregnancy outcomes. The effect size was very small (ε^2^ = 0.009), suggesting a negligible practical difference between the BMI groups.

Descriptive statistics are summarized in Table 6.

### 3.13. Maternal Knowledge in Relation to Age

To explore whether maternal age was associated with the level of knowledge regarding the impact of maternal overweight and obesity on pregnancy and infant outcomes, a Spearman rank correlation was calculated. A small but statistically significant positive correlation was observed between age and knowledge score (rho = 0.124, *p* < 0.001, *n* = 958). This shows that older participants tended to achieve slightly higher knowledge scores; however, the effect size was very small, implying that age explains only a minimal proportion of the variability in knowledge. Therefore, although the association is statistically significant—likely due to the large sample size—it is not clinically or practically meaningful.

### 3.14. Determinants of Maternal Knowledge

A multiple linear regression analysis was conducted to identify predictors of the overall knowledge score. The model was statistically significant (F(11,885) = 6.35, *p* < 0.001) but explained only a small proportion of the variance in knowledge (7.3%; adjusted R^2^ = 0.062).

Three variables remained statistically significant independent predictors of higher knowledge levels: current breastfeeding (β = 6.06, *p* = 0.004), participation in antenatal classes (β = 2.64, *p* = 0.007), and older age (β = 0.22 per year, *p* = 0.034), however the associated effect sizes were modest (Table 7).

Educational level, pregnancy status, and pre-pregnancy BMI category were not significant predictors in the adjusted model (all *p* > 0.05).

No issues with multicollinearity were observed (VIFs 1.01–1.05), and the Durbin–Watson statistic (2.16) indicated no autocorrelation of residuals, supporting the adequacy of model assumptions.

## 4. Discussion

### 4.1. Overall Level of Maternal Knowledge

Our study revealed substantial gaps in women’s knowledge regarding the impact of maternal overweight and obesity on pregnancy and infant outcomes. More than half of the participants demonstrated a low level of knowledge, while only 4% achieved a high score. This pattern is consistent with previous research, which repeatedly highlights inadequate awareness of weight-related risks among women of reproductive age. Studies conducted in diverse populations similarly report limited understanding of the consequences of excessive pre-pregnancy weight, insufficient recognition of recommended gestational weight gain, and low familiarity with evidence-based lifestyle guidelines [8,9,45,46].

### 4.2. Sociodemographic Factors and Health Literacy

The study cohort consisted of women aged 18–45 years, with a mean age of 32.2 years and most participants clustered around their early thirties. This age structure closely reflects the contemporary reproductive-age population in Poland and Europe, enhancing the generalizability of the findings [39,47,48]. Women in this age range are also typically more engaged in pregnancy planning and health information seeking, which may partially explain the moderate overall knowledge levels despite gaps identified in specific domains. The small positive correlation between maternal age and knowledge scores suggests that older women may have slightly greater awareness of the consequences of maternal overweight and obesity.

The vast majority of participants had higher education (85.1%), which is markedly higher than the national average among women of reproductive age. According to the recent GUS report, approximately half of Polish women aged 25–34 years have completed higher education [39,49]. This overrepresentation of highly educated respondents likely reflects the online recruitment strategy and may introduce selection bias. Knowledge scores increased consistently with educational level, with the highest awareness among women with higher education and the lowest among those with vocational training. This gradient is consistent with broader evidence indicating that health literacy tends to increase with educational level, influencing individuals’ ability to interpret health information, engage with preventive guidance, and navigate healthcare resources [10,50,51,52]. However, these conclusions should be interpreted with caution given the very small number of respondents in the primary and vocational education categories, which limits the statistical reliability and generalizability of comparisons involving these groups.

### 4.3. Reproductive Experience and Healthcare Exposure

A large proportion of participants were already mothers, and over half were currently breastfeeding. This reproductive profile is important for interpreting the results. Women with previous pregnancy and breastfeeding experience are more likely to have been exposed to antenatal education, pediatric visits, and postpartum counselling, which may partially explain their higher knowledge levels observed in this study. Breastfeeding itself emerged as an independent predictor of knowledge in the multivariable model. Interestingly, despite the fact that most of the sample consisted of women who had already been pregnant—and more than half were currently breastfeeding—the overall level of knowledge remained low to moderate. This is noteworthy, as these women had multiple opportunities to acquire information during antenatal care, postpartum visits, and routine interactions with healthcare professionals. One could therefore expect higher levels of awareness in this group. The finding suggests that existing antenatal and postnatal education may not sufficiently address or reinforce knowledge related to overweight and obesity in pregnancy. If women who have already been exposed to the healthcare system demonstrate only moderate knowledge, it is likely that women without any prior reproductive experience may be even less informed. However, because attendance is often influenced by socioeconomic and educational factors, the observed association may partly reflect underlying differences in health engagement rather than the direct effect of the classes themselves.

Conversely, women without previous reproductive experience constituted only a small proportion of the sample (5.5% planning a pregnancy and 5.3% in their first pregnancy). This limited representation may reduce the ability to generalize the findings to women who have not yet been pregnant—an important group, given that preconception knowledge is known to be suboptimal in many populations and strongly influences pregnancy behaviors.

### 4.4. Obstetric Characteristics and Pregnancy-Related Behaviors

In the present study, nearly 40% of all reported deliveries were completed by cesarean section, a proportion that closely mirrors national trends in Poland. According to recent reports, Poland is among the European countries with the highest cesarean section rates, with 43–47% of births delivered by C-section—significantly above the 10–15% threshold recommended by the World Health Organization [53,54]. The similarity between our findings and national statistics suggests that the study sample is representative in terms of obstetric outcomes.

The findings highlight that although the majority of women entered pregnancy with a normal BMI, excess body weight before conception was reported by more than one-third of respondents. This aligns with national estimates showing that nearly half of Polish women have excess body weight, a pattern driven by the steady increase in BMI with age [55]. Excessive pre-pregnancy body weight is a well-established risk factor for adverse maternal and neonatal outcomes, including gestational diabetes, hypertensive disorders, cesarean delivery, and long-term cardiometabolic complications in offspring [56,57,58].

A particularly concerning result is the very high proportion of pregnancies characterized by excessive gestational weight gain over 75% in the present sample, common across all BMI categories. This markedly exceeds international reports, where excessive GWG is about 45% [59]. Moreover, women with overweight demonstrated the highest likelihood of exceeding GWG recommendations. This pattern may reflect lower perceived risk or suboptimal counselling in this group, whereas women with obesity often receive more intensive monitoring and weight management guidance [11,16].

The study population demonstrated generally health-conscious behaviors during pregnancy, with very low rates of smoking and alcohol use and high adherence to recommended supplementation, particularly folic acid and vitamin D. A large proportion of respondents reported engaging in physical activity during pregnancy, and more than two-thirds attended antenatal classes. Current international guidelines [60,61,62], emphasize that regular moderate-intensity physical activity is safe for most pregnant women and is associated with multiple benefits, such as reduced risk of excessive gestational weight gain, gestational diabetes, hypertensive disorders, and improved psychological well-being. Higher participation in physical activity and antenatal classes may indicate that our sample included women who are generally more health-oriented and proactive in seeking pregnancy-related information.

Breastfeeding behaviors in our sample may also provide contextual insight into women’s overall health engagement. Although nursing was not the primary focus of this study, the high prevalence of breastfeeding intention and the considerable proportion of respondents who breastfed for ≥12 months suggest strong participation in perinatal health behaviors. Therefore, the high prevalence of nursing intention and the long breastfeeding durations observed in our sample may reflect greater involvement in perinatal education, which in turn could contribute to better knowledge regarding overweight and obesity in pregnancy [63,64]. However, not all studies confirm this relationship—for example, a large German cohort [65] found no significant association between maternal health literacy and breastfeeding behaviors.

Despite the overall moderate knowledge scores observed in this study, most respondents declared that they were aware of how maternal overweight or obesity may affect the course of pregnancy. This perceived awareness, however, did not consistently translate into accurate or comprehensive knowledge, as demonstrated by the detailed scoring results. Similar discrepancies between self-reported understanding and actual knowledge have been described in previous research, suggesting that women may overestimate their familiarity with weight-related pregnancy risks [8,9,46,50,66,67,68,69]. The notable proportion of participants who selected “uncertain” further highlights that, although many women believe they understand the consequences of excess maternal weight, their confidence in this knowledge is inconsistent.

Interestingly, women most frequently identified metabolic and hypertensive complications (GDM, gestational hypertension), whereas symptoms such as varicose veins, hemorrhoids, edema or constipation—although also more common in women with higher BMI due to increased venous pressure and reduced mobility—were mentioned less often [1,2,56,70,71]. This pattern suggests that participants may primarily associate overweight and obesity with major medical conditions, while being less aware of the increased risk of mechanical or functional symptoms. Women in our study demonstrated noticeably lower accuracy when identifying risks related to the child compared with maternal complications. While macrosomia, childhood obesity and metabolic consequences were recognized by many respondents, awareness of other important neonatal outcomes—such as shoulder dystocia, prematurity or congenital anomalies—was considerably less common [1,2,72,73,74,75]. This evidence implies that women may perceive the consequences of excess maternal weight primarily through the lens of its immediate maternal effects, while being less aware of the broader and well-established spectrum of fetal risks.

### 4.5. Skepticism Toward Weight-Based Health Information

The observed knowledge gaps may not solely reflect insufficient delivery of educational content, but may also partly indicate resistance to, or skepticism toward, weight-centered health messaging. Although free-text responses were limited in number, several participants expressed critical views regarding BMI-based risk assessment, questioned the causal role of overweight in adverse outcomes, or emphasized alternative determinants of health such as overall lifestyle, family habits, or non-weight-related indicators. These comments suggest that, for some women, weight-focused counseling may be perceived as overly simplistic, stigmatizing, or insufficiently individualized. Such attitudes could reduce receptiveness to educational messages, even when information is available, and may contribute to the persistence of knowledge gaps observed in this study. This illustrates the importance of not only improving the content of educational interventions, but also considering how messages are framed, communicated, and contextualized within maternity care.

### 4.6. Knowledge of Nutritional Recommendations During Pregnancy

Knowledge regarding recommended energy intake during pregnancy was highly inconsistent across all trimesters, with fewer than half of respondents correctly identifying the caloric requirements at any stage. A marked proportion of women either overestimated or underestimated the recommended increases, and many reported complete uncertainties. Similar patterns have been reported in another research. In a Polish study of 325 women of reproductive age, Kostecka et al. [76] found that although pregnant women were generally aware that caloric intake should increase across trimesters, the majority overestimated the recommended intake, particularly in the first and third trimester. This limited awareness is clinically relevant, as inappropriate assumptions about caloric requirements may contribute to unhealthy gestational weight gain patterns.

Understanding of the safety of weight reduction during pregnancy was mixed, with many women uncertain about whether intentional weight loss is recommended. Restricting energy intake for the purpose of weight loss is not considered appropriate during pregnancy, as it may compromise fetal growth and has not been demonstrated to reduce obesity-related risks [77,78]. Instead, guidelines focus on achieving appropriate gestational weight gain, optimizing diet quality, and promoting regular physical activity [5].

### 4.7. Sources of Information and Quality of Counselling

In our sample, the internet was the most frequently cited source of information, followed by obstetrician-gynecologists and midwives. This aligns with previous research demonstrating widespread internet use among pregnant women seeking health information. While online resources improve accessibility, their variable quality and reliability raise concerns; information found on the internet is often inaccurate [79,80,81,82].

Lifestyle counselling during pregnancy remains insufficient. Fewer than half of the respondents reported receiving dietary or physical activity advice, despite the important role of these behaviors in preventing excessive gestational weight gain and obesity-related complications. Similar observations have been made in qualitative studies, where women frequently describe counselling on gestational weight gain, nutrition and exercise as brief, unclear or inconsistent [83,84,85]. Barriers on the provider side—including limited consultation time, competing clinical priorities, discomfort initiating discussions about weight, and uncertainty about how best to support behavior change, and in some cases deficient provider knowledge regarding evidence-based lifestyle guidance—have also been documented and may contribute to the variability in counselling experiences [86,87,88,89]. What is interesting although most respondents with previous pregnancy experience reported receiving information about child health benefits of breastfeeding, far fewer recalled being informed about maternal health benefits.

### 4.8. Breastfeeding Perceptions

The overwhelmingly positive perception of breastfeeding among women with overweight or obesity in our sample is noteworthy, as it suggests that excess maternal weight is not commonly viewed as a barrier to lactation. This is encouraging given that physiological challenges such as delayed lactogenesis or lower confidence in breastfeeding ability have been more frequently reported in women with higher BMI in previous studies [22,23].

Most women who believed that infant feeding practices during the first six months influence later development, including metabolic programming, indicates a strong awareness of the long-term significance of early nutrition. This reflects public health message emphasizing the role of breastfeeding and early-life diet in shaping metabolic and immunological outcomes [90,91,92]. However, the presence of uncertainty among a notable minority of participants suggests that knowledge in this area is not universal.

### 4.9. Determinants of Maternal Knowledge and Implications for Educational Strategies

The multivariable model identified only three independent predictors of higher knowledge: current breastfeeding, participation in antenatal classes, and older age. Although the effect sizes were small, these findings suggest that ongoing engagement with maternal or infant health contexts—such as breastfeeding or structured antenatal education—may provide more opportunities for exposure to health information relevant to overweight and obesity in pregnancy. The overall explanatory power of the model was modest (adjusted R^2^ = 0.062), indicating that although these factors contribute to knowledge differences, a large proportion of the variability remains unexplained.

Based on the observed knowledge gaps, future educational efforts could focus on integrating concise, evidence-based information on gestational weight gain, nutrition, and metabolic risks into routine antenatal care. Antenatal classes may represent a useful platform for structured education; however, their content could be revised to place greater emphasis on metabolic health rather than general lifestyle advice. In addition, brief, standardized counseling messages delivered during routine prenatal visits, supported by accessible digital or printed materials, may help reinforce key concepts across different stages of the reproductive period. However, the limited explanatory power of the identified determinants suggests that gaps in maternal knowledge are unlikely to be addressed through educational efforts alone. Structural constraints within maternity care, such as limited consultation time and variability in counseling practices, as well as psychological factors including information overload, anxiety, or low perceived relevance of metabolic risks, may further limit the effectiveness of education. In addition, communicative barriers—such as inconsistent messaging across healthcare providers or the use of non-tailored information—may contribute to persistent misunderstandings despite frequent contact with maternity services. Taken together, these findings indicate that improving maternal health literacy is likely to require coordinated, multi-level approaches that extend beyond education alone and address how, when, and in what context information is delivered within maternity care.

## 5. Strengths and Limitations of the Study

To the best of our knowledge, this is the first study conducted in Poland that comprehensively evaluates women’s knowledge regarding maternal overweight and obesity and their potential adverse effects on pregnancy and infant outcomes. The research was performed in a large, nationwide sample of adult women, enhancing the generalizability of the findings. Importantly, our results highlight a consistently low level of awareness in this area, underscoring a critical gap in public health education.

Although this study has several limitations that should be acknowledged.

First, while the questionnaire was developed based on current evidence and previously published tools, no validated Polish instrument specifically designed to assess women’s knowledge of overweight and obesity in pregnancy is currently available. Therefore, the lack of a formally validated tool may limit the comparability of our results with studies from other populations. Nevertheless, the internal consistency of the survey was acceptable, which partially mitigates this limitation.

Second, the use of online recruitment may introduce selection bias. Women who are more digitally active, younger, or with higher educational attainment may have been more likely to participate, potentially affecting the distribution of knowledge scores. However, the large sample size improves the robustness of the findings, the possibility of overrepresentation of certain demographic groups cannot be fully excluded.

## 6. Conclusions

This study reveals that many women still lack a solid understanding of how maternal overweight and obesity affect pregnancy and infant health. Even among a relatively well-educated, health-engaged population with prior reproductive experience, important knowledge gaps were observed. These findings suggest that current antenatal and postnatal care may not adequately address or provide evidence-based information on weight-related risks.

Factors associated with higher knowledge—such as breastfeeding, attending antenatal classes, and older age—seem to reflect greater exposure to the healthcare system rather than a deeper understanding of the underlying metabolic clinical implications. Importantly, these variables together explained only a small part of the variation in knowledge scores, highlighting the limited explanatory power of the measured determinants and the complexity of maternal health literacy.

Taken together, these findings suggest that educational interventions, while necessary, may not be sufficient on their own to address existing knowledge gaps. At the same time, there remains is a clear need for better, more consistent and easier-to-access education on maternal overweight and obesity throughout the entire reproductive period—starting before conception and continuing during pregnancy and postpartum. Topics such as gestational weight gain, nutrition, metabolic risks, and possible outcomes for the newborn should be communicated more clearly and more routinely, both in clinical practice and in public health initiatives. In addition, improving maternal knowledge is likely to require addressing structural, communicative, and psychological barriers within maternity care. Future strategies may, therefore, benefit from integrating educational efforts with broader system-level approaches to support effective communication and personalized counselling across the reproductive continuum.

## Figures and Tables

**Table 1:** Participants’ characteristics.

Sociodemographic Variable		*n*/*N*	%
Age (years)	18–25	62/958	6.5
	26–30	304/958	31.7
	31–35	373/958	38.9
	36–40	174/958	18.2
	41–45	45/958	4.7
Residence	Rural	230/958	24
	Urban, <50,000 residents	123/958	12.8
	Urban, 50,000–149,999 residents	136/958	14.2
	Urban, 150,000–499,999 residents	166/958	17.3
	Urban, ≥500,000 residents	303/958	31.6
Education	Primary education	3/958	0.3
	Vocational education	10/958	1
	Secondary education	130/958	13.6
	Higher education	815/958	85.1
Marital status	Single	30/958	3.1
	Informal relationship	157/958	16.4
	Married	761/958	79.4
	Divorced/Separated	9/958	0.9
	Widowed	1/958	0.1

Data are presented as *n*/*N* (%), where *n* denotes the number of respondents in a given category and *N* denotes the total number of respondents with available data for that variable.

**Table 2:** Obstetric variables.

Variable		*n*/*N*	%
Reproductive status	Planning pregnancy	53/958	5.5
	First pregnancy	51/958	5.3
	Already a mother, currently breastfeeding	498/958	52
	Already a mother, currently not breastfeeding	241/958	25.2
	Subsequent pregnancy (already has child/children)	87/958	9.1
	Subsequent pregnancy, currently breastfeeding	28/958	2.9
Previous pregnancies	0	52/958	5.4
	1	427/958	44.6
	2	297/958	31
	3	117/958	12.2
	4	47/958	4.9
	5 and more	18/958	1.9
Mode of delivery	Vaginal delivery	734/1218	60.3
	Emergency cesarean section	284/1218	23.3
	Planned cesarean section	200/1218	16.4
Gestational age	<22 weeks (miscarriage)	215/878	24.5
	37–40 weeks	370/878	42.1
	40–42 weeks	279/878	31.8
	>42 weeks	14/878	1.6

Data are presented as *n*/*N* (%), where *n* denotes the number of respondents in a given category and *N* denotes the total number of respondents with available data for the specific variable.

**Table 3:** Maternal weight status before and during pregnancy.

Variable		*n*/*N*	%
Pre-pregnancy BMI	Underweight	52/906	5.7
	Normal weight	557/906	61.5
	Overweight	188/906	20.8
	Obesity	109/906	12.0
Pregnancies affected by pre-pregnancy overweight/obesity	1	173/297	58.2
	2	88/297	29.6
	3	26/297	8.8
	4	9/297	3.0
	5	1/297	0.3
Gestational weight gain	Adequate	317/1284	24.7
	Excessive	967/1284	75.3

Data are presented as *n*/*N* (%), where *n* denotes the number of respondents in a given category and *N* denotes the total number of respondents with available data for the specific variable.

**Table 4:** Distribution of gestational weight gain adequacy across pre-pregnancy BMI categories (based on the first reported pregnancy per participant).

BMI Category	Adequate GWG *n* (%)	Excessive GWG *n* (%)	Total (*N*)
Underweight	43 (91.5)	4 (8.5)	47
Normal weight	412 (78.8)	111 (21.2)	523
Overweight	110 (62.1)	67 (37.9)	177
Obesity	74 (72.5)	28 (27.5)	102
Total	639 (75.3)	210 (24.7)	849

Data are presented as *n* (%); percentages are calculated within BMI categories; *N* denotes the total number of respondents with available data for the specific variable.

**Table 5:** Breastfeeding practices.

Category		*n*/*N*	%
Intention to breastfeed	Yes/have breastfed	904/958	94.4
	No	39/958	4.1
	Undecided	15/958	1.6
Duration of breastfeeding	1 month or less	50/693	7.2
	2–3 months	60/693	8.7
	4–6 months	63/693	9.1
	7–12 months	98/693	14.1
	More than 1 year	226/693	32.6
	More than 2 years	196/693	28.3

Data are presented as *n*/*n* (%), where *n* denotes the number of respondents in a given category and *n* denotes the total number of respondents with available data for the specific variable.

**Table 6:** Maternal knowledge scores across key participant characteristics.

Group	Category	*N*	Mean (%)	Median (%)	SD	Min	Max
Total sample	-	958	57.6	57.9	13.6	18.4	89.5
Educational level	Primary	3	50.0	47.4	12.1	39.5	63.2
	Vocational	10	38.2	434	13.1	18.4	55.3
	Secondary	130	53.0	52.6	13.6	18.4	81.6
	Higher	815	58.6	60.5	13.4	18.4	89.5
Participation in antenatal classes	No	288	55.2	55.3	14.1	18.4	86.8
	Yes	609	59.0	60.5	13.1	18.4	89.5
Reproductive status	Planning pregnancy	53	53.9	52.6	14.6	23.7	78.9
	First pregnancy	51	54.3	55.3	14.6	18.4	81.6
	Has child—not breastfeeding	328	56.2	55.3	13.9	18.4	86.8
	Has child—breastfeeding	526	59.2	60.5	13.1	23.7	89.5
BMI category	Underweight	52	56.6	55.3	13.6	28.9	81.6
	Normal weight	557	57.6	60.5	13.4	18.4	89.5
	Overweigh	188	57.9	57.9	13.7	21.1	84.2
	Obesity	109	59.8	60.5	14.0	23.7	86.8

Values are presented as mean (%), median (%), standard deviation (SD), minimum (Min), and maximum (Max) knowledge scores.

**Table 7:** Independent Predictors of Maternal Knowledge Score (Adjusted Model *).

Predictor	β	SE	*p*-Value
Current breastfeeding	6.06	2.09	0.004
Attended antenatal classes	2.64	0.98	0.007
Maternal age	0.22	0.10	0.034

* The model was adjusted for maternal age, educational level, pregnancy status (pregnant vs. non-pregnant), pre-pregnancy BMI category, current breastfeeding status, and participation in antenatal classes.

## Data Availability

The data presented in this study are available from the corresponding author upon reasonable request. The data are not publicly available due to privacy concerns related to sensitive health information collected in an anonymous survey.

## Supplementary Materials

The following supporting information can be downloaded at: https://www.mdpi.com/article/10.3390/nu18020203/s1, Figure S1: Directed acyclic graph (DAG) illustrating the assumed causal relationships between sociodemographic factors, reproductive characteristics, healthcare exposure, and maternal knowledge. Arrows indicate assumed directional relationships between variables. The diagram was created using DAGitty (https://dagitty.net, accessed on 29 December 2025); Figure S2: Awareness of how maternal overweight or obesity may affect the course of pregnancy. Numbers indicate the absolute number of respondents; Figure S3: (A) Knowledge of caloric requirements in the first trimester. Numbers indicate the absolute number of respondents; (B) Knowledge of caloric requirements in the second trimester. Numbers indicate the absolute number of respondents; (C) Knowledge of caloric requirements in the third trimester. Numbers indicate the absolute number of respondents; Figure S4: Perceptions of intentional weight reduction during pregnancy. Numbers indicate the absolute number of respondents; Figure S5: Sources of pregnancy-related health information (multiple responses allowed); Figure S6: Participants’ responses on whether overweight/obese women can breastfeed. Numbers indicate the absolute number of respondents; Figure S7: Perceived impact of infant feeding in the first 6 months on later development (“metabolic programming”). Numbers indicate the absolute number of respondents; Table S1: Pregnancy management and health behaviors; Table S2: Dietary and physical activity counselling received during pregnancy; Table S3: Information received about breastfeeding benefits.
