# Supplementary material for: Knowledge Gaps Regarding Overweight and Obesity in Pregnancy: A Cross-Sectional Study Among Polish Women
Source: Nutrients. 2026 Jan 8;18(2):203. doi: 10.3390/nu18020203 (PMC12844759; doi:10.3390/nu18020203)
Supplement: Supplementary file 1 [file nutrients-18-00203-s001.zip › nutrients-4066212-supplementary.pdf]

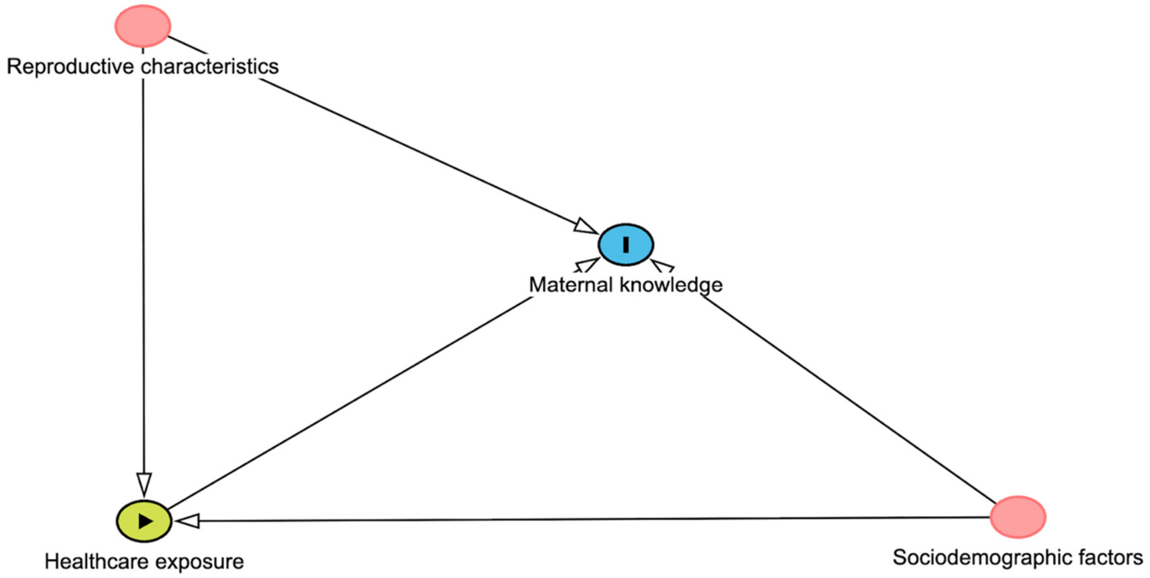

Supplementary Figure S1. Directed acyclic graph (DAG) illustrating the assumed causal relationships between sociodemographic factors, reproductive characteristics, healthcare exposure, and maternal knowledge. Arrows indicate assumed directional relationships between variables. The diagram was created using DAGitty (https://dagitty.net)

Supplementary Table S1. Pregnancy management and health behaviors.

Category		n/N	%
**Physical activity during pregnancy**	Yes	573/903	63.4
	No	330/903	36.6
**Antenatal class attendance**	Yes	609/906	67.2
	No	297/906	32.8
**Alcohol use during pregnancy**	Regular	0/906	0
	Occasional	2/906	0.2
	None	904/906	99.8
**Smoking in pregnancy**	Regular	14/906	1.5
	Occasional	11/906	1.2
	None	881/906	97.2
**Supplement and medication use**	Pregnancy-specific supplements	840/906	92.7
	Folic acid	691/906	76.3
	Vitamin D	549/906	60.6
	Antiallergic drugs	45/906	5.0
	Urinary tract infections medications	159/906	17.6
	Reproductive tract infection medications	163/906	18.0
	Antibiotics	161/906	17.8
	Thyroid medications	294/906	32.5
	Antihypertensive medications	89/906	9.8
	Insulin resistance/diabetes medications	98/906	10.8

	Anticoagulation therapy	153/906	16.9
	Antidepressants	23/906	2.5
	Other medications	99/906	10.9

Data are presented as *n*/*N* (%), where *n* denotes the number of respondents in a given category and *N* denotes the total number of respondents with available data for the specific variable.

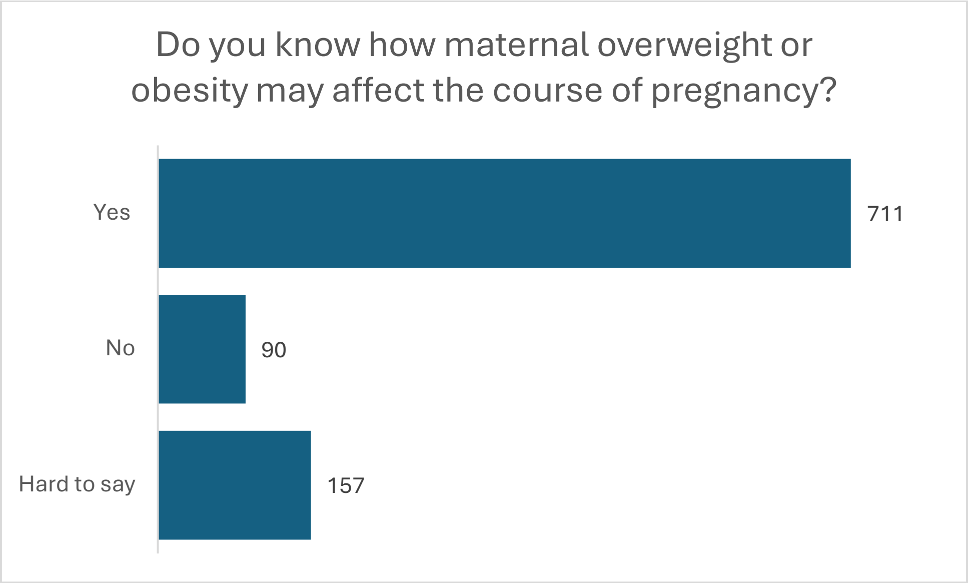

Supplementary Figure S2. Awareness of how maternal overweight or obesity may affect the course of pregnancy. Numbers indicate the absolute number of respondents.

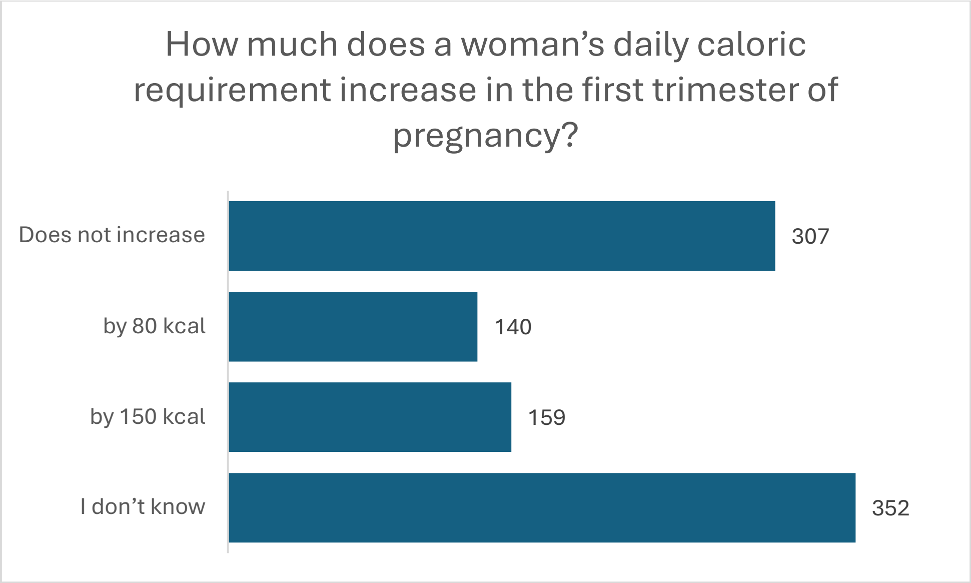

Supplementary Figure S3A. Knowledge of caloric requirements in the first trimester. Numbers indicate the absolute number of respondents.

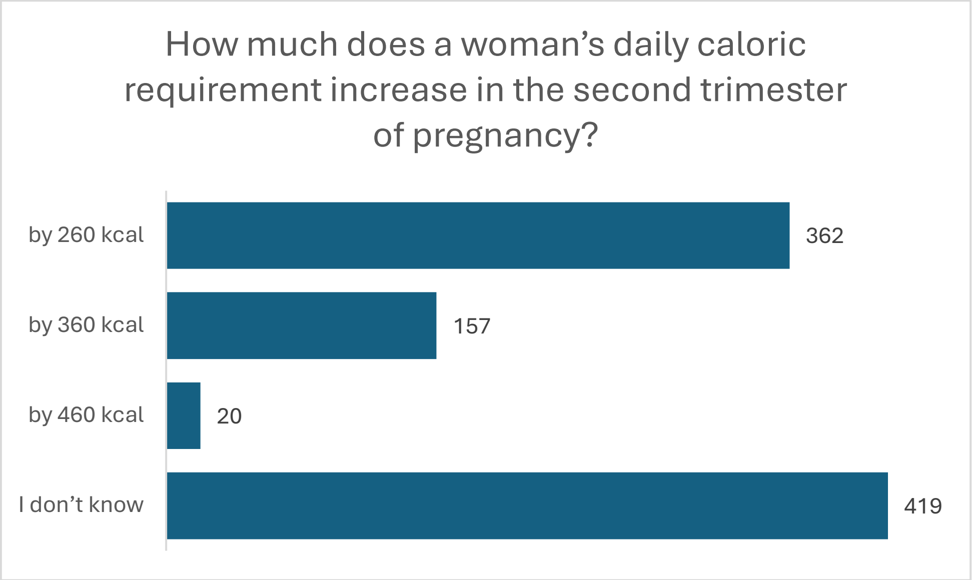

Supplementary Figure S3B. Knowledge of caloric requirements in the second trimester. Numbers indicate the absolute number of respondents.

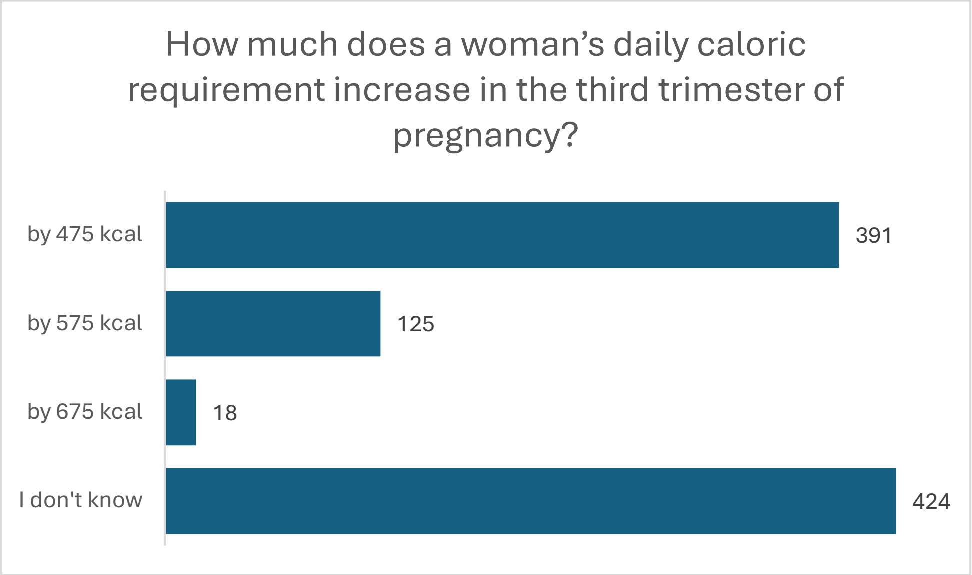

Supplementary Figure S3C. Knowledge of caloric requirements in the third trimester. Numbers indicate the absolute number of respondents.

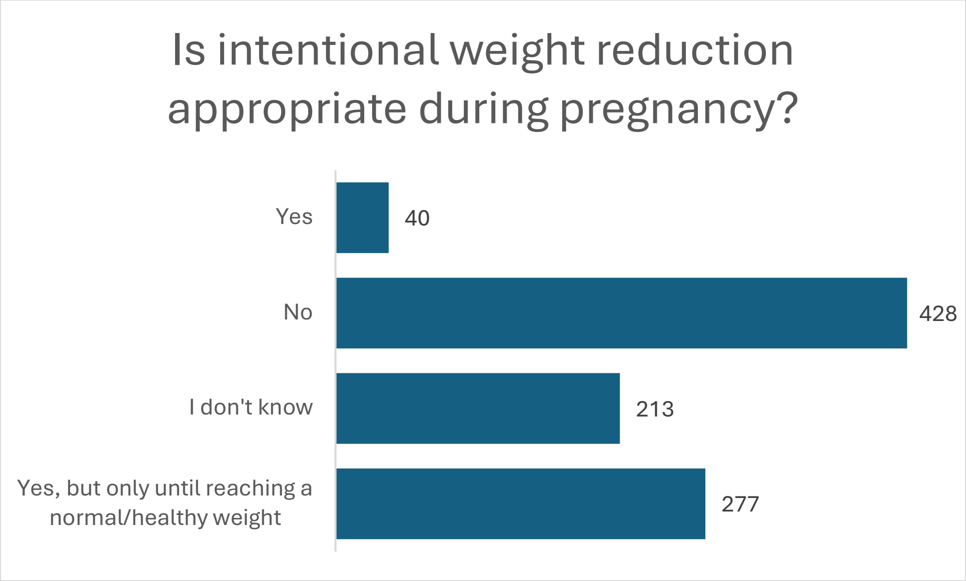

Supplementary Figure S4. Perceptions of intentional weight reduction during pregnancy. Numbers indicate the absolute number of respondents.

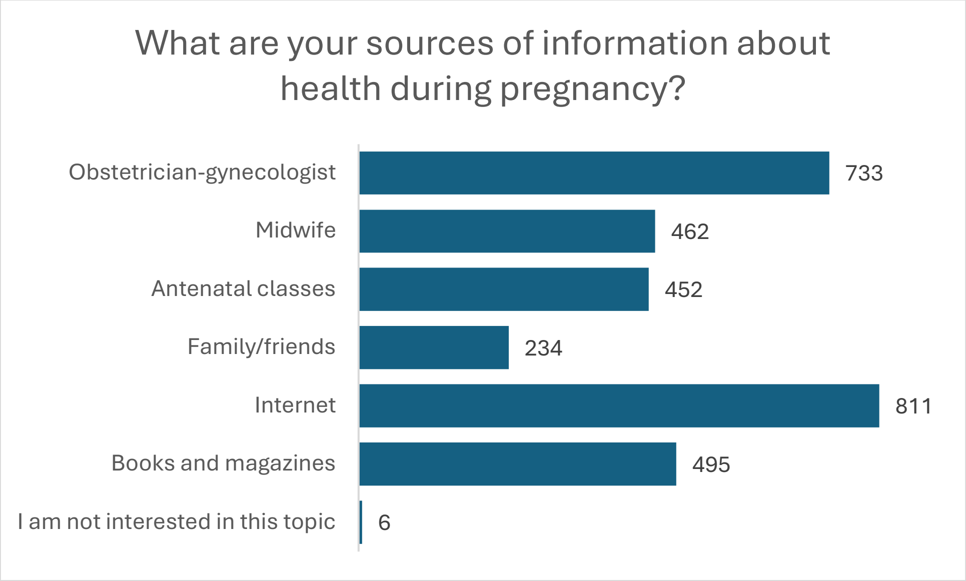

Supplementary Figure S5. Sources of pregnancy-related health information (multiple responses allowed).

Supplementary Table S2. Dietary and physical activity counselling received during pregnancy

Category		n/N	%
**Dietary counselling during pregnancy**	Yes	347/899	38.6
	No	507/899	56.4
	Don't remember	45/899	5.0
**Physical activity counselling during pregnancy**	Yes	399/899	44.4
	No	445/899	49.5
	Don't remember	55/899	6.1

Data are presented as n/N (%), where n denotes the number of respondents in a given category and N denotes the total number of respondents with available data for the specific variable.

Supplementary Table S3. Information received about breastfeeding benefits.

Category		n/N	%
**Told about benefits for the child**	Yes	622 /895	69.5
	No	225 /895	25.1
	Don't remember	48 /895	5.4
**Told about benefits for the mother**	Yes	479/896	53.5
	No	352/896	39.3
	Don't remember	65/896	7.3

Data are presented as *n*/*N* (%), where *n* denotes the number of respondents in a given category and *N* denotes the total number of respondents with available data for the specific variable.

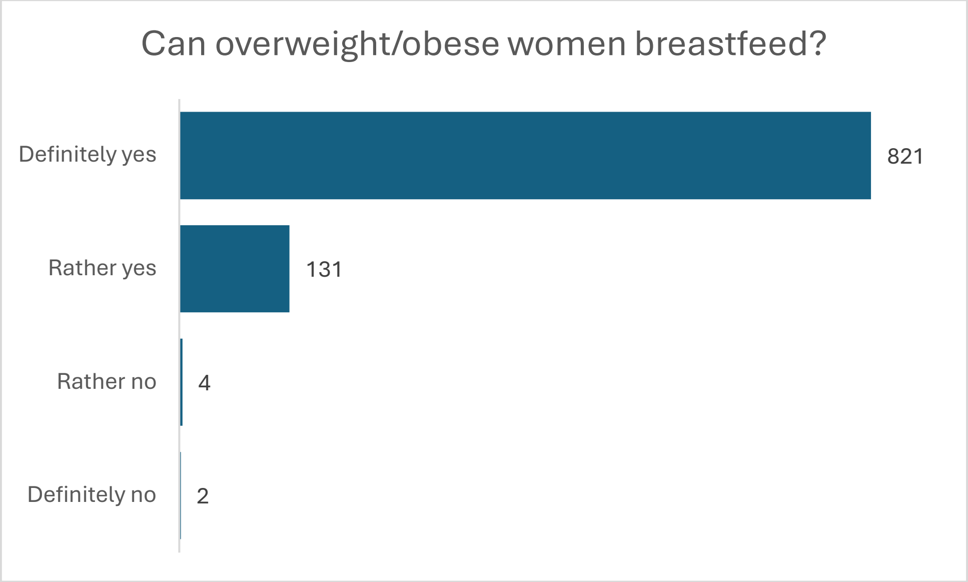

Supplementary Figure S6. Participants' responses on whether overweight/obese women can breastfeed. Numbers indicate the absolute number of respondents.

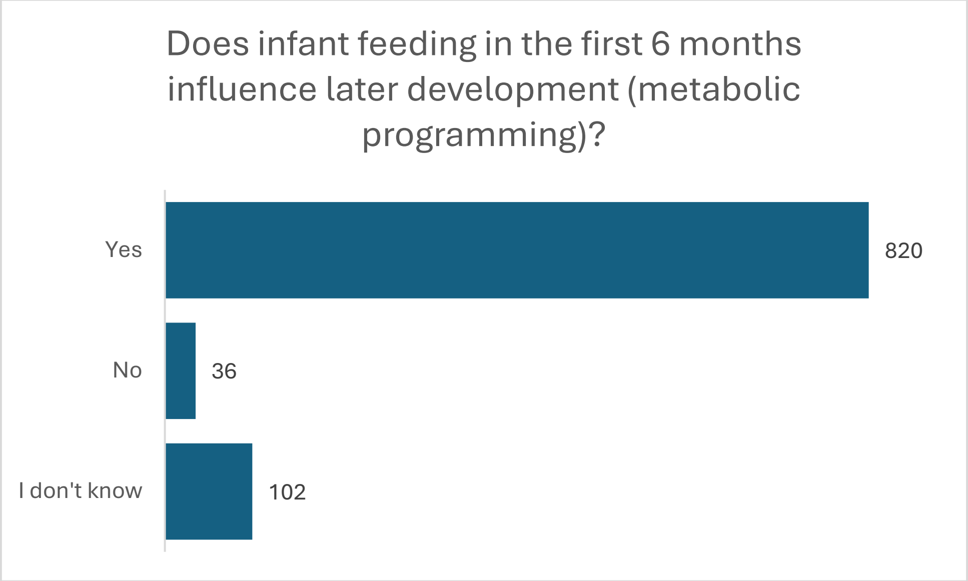

Supplementary Figure S7. Perceived impact of infant feeding in the first 6 months on later development ("metabolic programming"). Numbers indicate the absolute number of respondents.
